# Un mélanome choroïdien de découverte fortuite sur un œil amblyope

**DOI:** 10.11604/pamj.2024.47.79.42795

**Published:** 2024-02-21

**Authors:** Taha Boutaj, Manal Tabchi

**Affiliations:** 1 Service d'Ophtalmologie “A”, Centre Hospitalier Universitaire Ibn Sina, Hôpital des Spécialités, Mohammed V University, Rabat, Maroc

**Keywords:** Mélanome, choroïde, amblyope, échographie oculaire, IRM orbitaire, Melanoma, choroid, amblyopic, ocular ultrasound, orbital MRI

## Abstract

We report the case of a 37-year-old patient with profound amblyopia of the left eye from childhood, who attended a consultation as part of diabetic retinopathy screening following a recent diagnosis of diabetes. Ophthalmological examination revealed a visual acuity of 10/10 in the right eye and 1/10 in the left eye. The anterior segment was normal. Fundus examination of the left eye showed a large achromatic mass in the temporal retina, surrounded by a serous retinal detachment with interpapillomacular folds (A). Ocular ultrasound revealed a hyperechoic mushroom-shaped choroidal process with vascularity on colour Doppler ultrasound (B). Orbital Magnetic Resonance Imaging (MRI) revealed left circumscribed intra-orbital process, with mean signal intensity after gadolinium injection (C). After a multidisciplinary meeting, the diagnosis of choroidal melanoma was strongly suspected. Assessment of extent including brain MRI, thoraco-abdominopelvic Computed Tomography (CT) scan and abdominal ultrasound was normal. Given the size of the mass, conservative treatment was not possible. The patient underwent an enucleation and was referred to the oncology department for further management, after anatomopathological confirmation of the surgical specimen.

## Image en médecine

Nous rapportons le cas d'un patient de 37 ans, amblyope profond de l'œil gauche depuis l'enfance non suivi, qui consulte dans le cadre du dépistage de la rétinopathie diabétique à la suite du diagnostic récent d'un diabète. L'examen ophtalmologique du patient retrouve une acuité visuelle à 10/10^e^au niveau de l'œil droit, et de 1/10 à l'œil gauche. Le segment antérieur est normal. Le fond d'œil trouve à l'œil gauche une volumineuse masse achrome bourgeonnante dans la rétine temporale, entourée d'un décollement séreux rétinien avec la présence de plis en inter papillo-maculaire (A). L'échographie oculaire retrouve un processus choroïdien hyperéchogène en champignon, vascularisé au Doppler (B). L'imagerie par résonance magnétique (IRM) orbitaire objective un processus intra orbitaire gauche limité, de signal d'intensité moyenne après injection de gadolinium (C). Après réunion pluridisciplinaire, le diagnostic de mélanome choroïdien a été fortement suspecté. Un bilan d'extension comprenant une IRM cérébrale, une tomodensitométrie (TDM) thoraco-abdomino-pelvienne et une échographie abdominale est revenu normal. Devant la volumineuse masse, un traitement conservateur n'a pas pu être réalisé. Le patient a donc été énucléé, puis adressé en service d'oncologie pour complément de prise en charge après confirmation anatomo-pathologique de la pièce opératoire.

**Figure 1:**
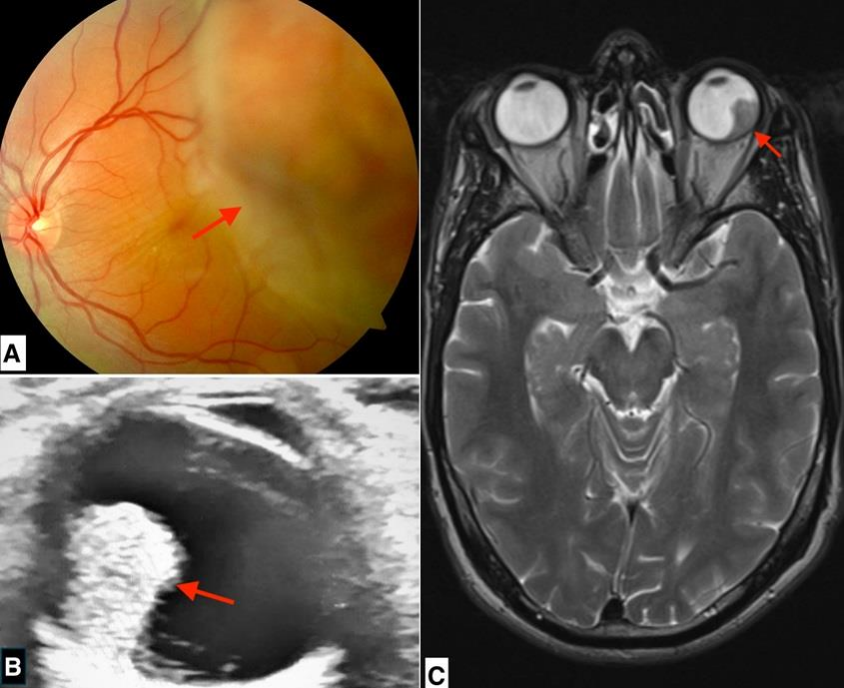
A) fond d'œil: volumineuse masse achrome bourgeonnante dans la rétine temporale, entourée d'un décollement séreux rétinien avec la présence de plis en inter papillo-maculaire (flèche); B) échographie oculaire: processus choroïdien hyperéchogène en champignon, vascularisé au Doppler (flèche); C) IRM orbitaire: processus intra orbitaire gauche limité, de signal d'intensité moyenne après injection de gadolinium (flèche)

